# β-glucan nanoparticles alleviate acute asthma by suppressing ferroptosis and DNA damage in mice

**DOI:** 10.1007/s10495-024-02013-9

**Published:** 2024-09-21

**Authors:** Bassam W. Ebeed, Islam Ahmed Abdelmawgood, Mohamed A. Kotb, Noha A. Mahana, Ayman Saber Mohamed, Marwa A. Ramadan, Abeer Mahmoud Badr, Manar Nasr, Osama Mohsen Qurani, Reem Mohamed Hamdy, Nada Yasser Abd El-Hakiem, Mariam Khaled Fahim, Mariam Morris Fekry, Jehane I. Eid

**Affiliations:** 1 Zoology Department, Faculty of Science, Cairo University, Giza, 12613 Egypt; 2 Department of Laser Application in Metrology Photochemistry and Agriculture, National Institute of Laser Enhanced Science NILES Cairo University, Giza, Egypt; 3 Faculty of Biotechnology, October University for Modern Sciences and Arts, 6th of October, Egypt

**Keywords:** Beta-glucan nanoparticles, Ovalbumin, Acute asthma, Oxidative stress, Inflammation, Ferroptosis

## Abstract

Asthma is a severe respiratory disease marked by airway inflammation, remodeling, and oxidative stress. β-Glucan (BG), a polysaccharide constituent of fungal cellular structures, exhibits potent immunomodulatory activities. The investigational focus was on the anti-asthmatic and anti-ferroptotic properties of beta-glucan nanoparticles (BG-NPs) in a murine model of allergic asthma induced by ovalbumin (OVA). BG was extracted from Chaga mushrooms (*Inonotus obliquus*), and its BG-NPs were characterized utilizing techniques including FT-IR, UV visible spectroscopy, zeta potential analysis, DLS, XRD, and TEM. The Balb/C mice were allocated into five groups: control, untreated asthmatic, dexamethasone (Dexa)-treated (1 mg/kg), BG-treated (100 mg/kg), BG-NPs-treated (45 mg/kg), and BG-treated (100 mg/kg). Treatment with BG-NPs markedly diminished the entry of inflammatory cells into the respiratory passage, serum IgE concentrations, DNA damage, and markers of oxidative stress through the reduction of malonaldehyde (MDA) levels and enhancing the levels of reduced glutathione (GSH), glutathione peroxidase (GPx), superoxide dismutase (SOD), and catalase (CAT). Furthermore, BG-NPs reduced iron deposition and promoted the transcriptional activity of the GPx4 gene in pulmonary cells, attenuating ferroptosis. The results demonstrated that BG-NPs reduced asthma by inhibiting oxidative stress, inflammation, DNA damage, and ferroptosis. Our results suggest that BG-NPs could be used as potential treatments for allergic asthma.

**Graphical abstract:**

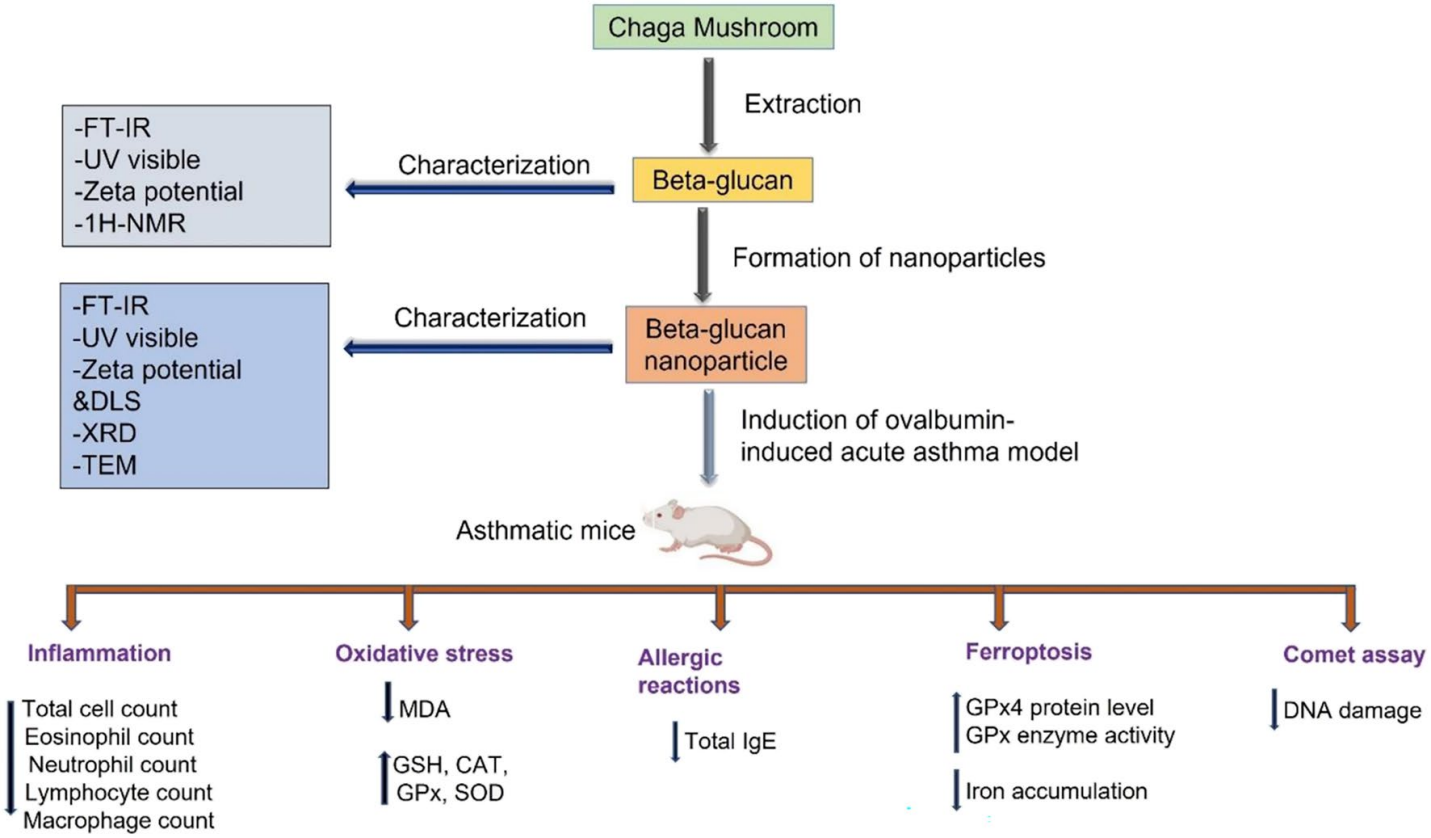

## Introduction

Asthma is a prevalent pulmonary disorder wherein immune response dysregulation causes chronic airway inflammation. The condition affects approximately 5 percent of the global population [1, 2]. The multilayered pathophysiology of asthma includes the interaction of mast cells, eosinophils, and activated T helper lymphocytes with epithelial cells and airway smooth muscle cells. This complex interplay leads to the release of proinflammatory cytokines and lipid mediators [3, 4]. It has been proposed that the primary factor in the onset and advancement of this condition is the disequilibrium between Th1 and Th2 responses. In patients with asthma, there is an overproduction of cytokines of Th2-type, such as IL-4, IL-5, and IL-13. These cytokines play a role in the production of IgE antibodies, eosinophil migration and longevity, excessive mucin synthesis, increased sensitivity of the airways, tissue remodeling, and the creation of reactive oxygen species (ROS) [5–7]. Moreover, eosinophil movement to the airway and the production of ROS that may damage the airway are additional causes of oxidative stress in asthma. Exposure of the lungs to oxidative stress can lead to Increased generation of ROS. This diminishes the antioxidant activity, raises inflammatory mediator secretion, and results in goblet cell hyperplasia [8–10]. Furthermore, ferroptosis is a form of cell death that depends on iron. The decreased scavenging action of glutathione peroxidase 4 (GPx4) and the excessive buildup of ROS associated with iron ions in the cells are the primary causes of ferroptosis [11]. Ferroptosis has been linked to some respiratory disorders, including eosinophilic asthma [12].

For most asthma patients, corticosteroids and anti-leukotrienes are effective treatments [13]. However, one of the main characteristics of patients with severe asthma is corticosteroid insensitivity. Despite the prolonged and frequent use of systemic, high-dose corticosteroids, these individuals have higher pulmonary oxidative stress and inflammation levels, which contribute to weakened lung function and persistent exacerbations [14].

Nanotechnology shows a lot of benefits in the medical field by giving a chance to use many components with lower doses with the same Or better efficiency, perfume many methods to carry the drug to its target with a low dose which gives us an advance to reduced toxicity of many drugs [15]. Medicinal compounds derived from natural sources have been effective in disease treatment over the years, proving an indispensable repertoire of promising therapeutic agents [16]. Mushrooms are well-known for their medical uses [17]. They are thought to possess numerous therapeutic benefits, such as antitumoral, antioxidant, immunomodulatory, hepatoprotective, and numerous additional features [18]. One of the active components with a high molecular weight in Chaga mushrooms is β-glucan (BG) [19–21]. BG extracted from fungi or yeast has been demonstrated to have immunomodulatory activities [22], and traditionally produced BG as medications have been restricted due to their enormous molecular weight as polysaccharides [23]. Consequently, BG-NPs were prepared to reduce the size of natural polysaccharides to nanoscale dimensions. The current study aims to elucidate the potential therapeutic effects of BG-NPs on oxidative stress, inflammation, and ferroptosis associated with asthma.

## Materials and methods

### BG extraction from Chaga mushroom

BG extraction was performed using the alkali extraction protocol [24] described. Briefly, 100 g of Chaga powder (Chi chaga, Canada) was solubilized in 1 L of sodium carbonate-bicarbonate buffer (pH 10), followed by stirring at 45 °C for 30 min and centrifugation at 15000 × g for 12 min. Subsequent to the retrieval of the supernatant, pH was adjusted to 4.5 using 2M hydrochloric acid (HCl). Following that, proteins were isolated by centrifuging at 15,000 × g for 20 min at 4 °C. To deactivate any enzymatic activity, the supernatant was heated to 100 °C for 10 min, then allowed to return to ambient temperature, and mixed in a 1:1 ratio with 50 mL of isopropanol. After settling the mixture overnight, BG was separated by centrifugation at 3000 rpm for 10 min. Finally, the supernatant was discarded, leaving behind an air-dried pellet that weighed 6 g.

### BG nanoparticles (BG-NPs) synthesis

BG-NPs were synthesized according to the technique outlined by [25] with some modifications. β-Glucan powder was dissolved in 2% NaOH (2 mg/ml) and stirred for 1 h at 90 °C. A dilute 1% acetic acid solution was added gradually till complete precipitation of BG-NPs. The suspension will be further stabilized by dropwise addition of tripolyphosphate (TPP) at a concentration of 0.8 mg/ml with continuous stirring. The mixture was stirred for 30 min at room temperature, and the β-glucan nanoparticles were collected by centrifugation. The type of nanoparticles is nano-reduction.

### Characterization of BG and BG-NPs

#### Fourier transform infrared (FT-IR) spectroscopy

BG and BG-NPs powders were mixed with potassium bromide and examined using a Nicolet 380 FT-IR spectrophotometer adjusted at the spectral range of 500–4000 cm^−1^.

#### UV/Vis spectroscopy

BG and BG-NPs were solubilized in distilled water, and a UV/Vis spectrophotometer (Spectro UV-500) was utilized to evaluate their absorbance over a wavelength range of 200–500 nm.

#### X-ray powder diffraction (XRD)

The integrated nanoparticles were studied for deciding the design creation and the translucent stage utilizing an X-beam diffractometer (XRD, D8-Find, Bruker, with CuKα radiation (1.5418 Å), Madison, WI, USA) working at a current of 40 MA, voltage of 40 kV and step filter 0.01°. In any case, the dried samples should be ready before estimation, tests processing utilizing a basic planetary ball factory (LZQM0.4L, Shicheng Desert spring Mineral Gear Assembling Co., Ltd.) in which ball factory of treated steel of 0.1 cm breadth put in processing measure with tests for 1 h at 1500 rpm [26].

#### Transmission *electron* microscopy (TEM)

Samples of BG and BG-NPs were placed on grids, dried, and observed for morphology and size using a transmission electron microscope (JEOL JEM-1400 TEM) at an appropriate magnification.

#### Zeta potential and dynamic light scattering (DLS)

Samples of BG and BG-NPs were dissolved in distilled water, and their zeta potential and particle size were measured using a Particle Sizing System (NICOMP) equipped with a zeta potential analyzer.

### Experimental animals

A total of 40 male BALB/c mice weighing 20 to 25 g were obtained from the National Research Center (NRC) and housed in the animal house facility of the Faculty of Science, Cairo University. The mice were housed in an environment adjusted to include a cycle of 12 h of light–dark. They were provided unrestricted access to food and water ad libitum. Prior to the beginning of the experiment, the mice were allocated to acclimatize for 1 week.

### Acute toxicity test (LD50)

The lethal dose (LD50) of BG-NP was determined following the procedure of Chinedu et al. [26]. The mice were subjected to overnight fasting before being divided into four groups (n = 2). Following the intraperitoneal injection, the animals were monitored at 1 h intervals at the beginning and then every 2 h over the next 24 h. After intraperitoneal injections of BG-NPs at doses 10, 100, 300, and 600 mg/kg, the animals were monitored for behavioral changes, including fatigue, semi-solid stools, excessive saliva production, and loss of appetite. The dose selected for the study was one-tenth of the LD 50.

$$LD 50 = \frac{The highest dose that did not result in death + \,The lowest dose that resulted in death }{2}$$

$$LD 50 = \frac{300 + 600}{2} = 450 mg/kg$$

A dose of 45 mg/kg was selected for BG-NPs.

### Experimental design

#### Ovalbumin-induced asthma model

The acute allergic asthma model was developed using OVA following a slightly modified method delineated by Hsu et al. [27]. The mice (five groups, n = 8/group): a control group (Control), an ovalbumin group (OVA), a group treated with reference drug dexamethasone (Dexa), a B-glucan group (BG), and a group treated with B-glucan nanoparticles (BG-NPs). The induction phase of the model consists of two primary stages: sensitization and challenge. For asthma induction, mice were injected intraperitoneally containing 20 µg OVA (Sigma Aldrich, USA) and 2 mg aluminum hydroxide gel (Al (OH)_3_) (Sigma Aldrich) on days 0 and 7. From days 14 to 16, all groups except the control one was exposed to inhalation of 5% OVA nebulized in phosphate buffer for 30 min, which was done one hour after the drug injections.

#### Treatment protocol

**Fig. 1:**
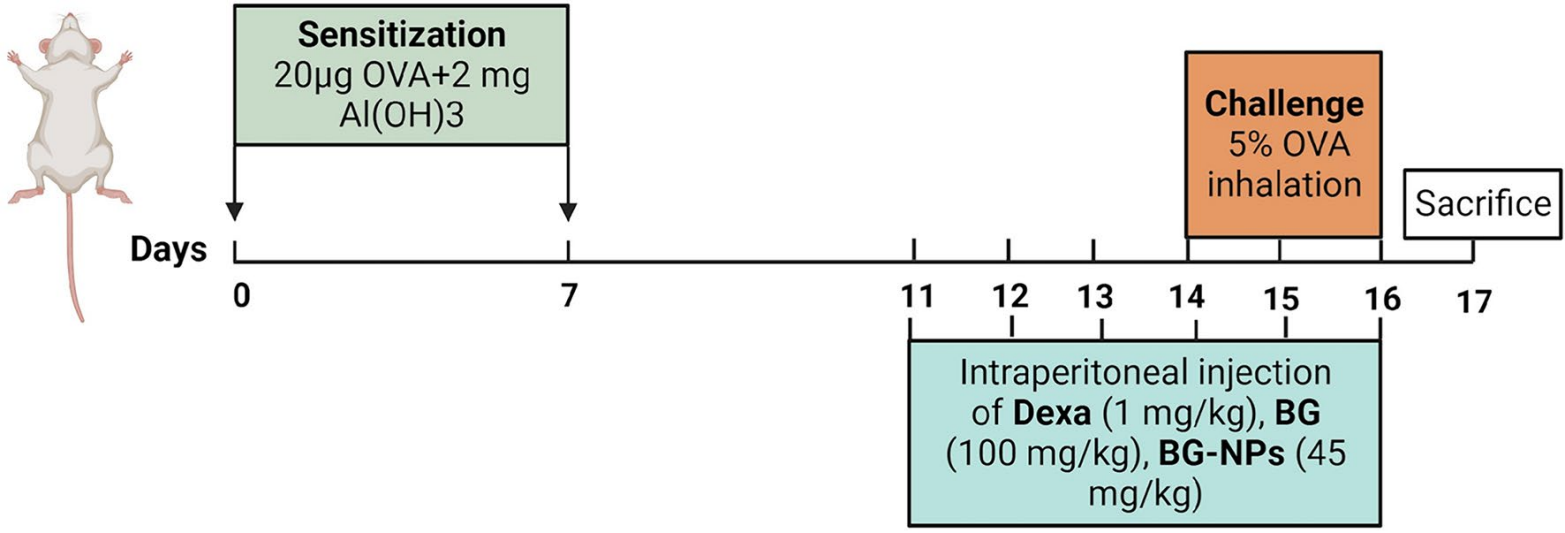
Schematic showing the steps involved in BG-NPs treatment and establishing the experimental model of acute asthma

During days 11 to 16, the respective drug doses were administrated intraperitoneally to treated groups: Dexa at a dosage of 1 mg/kg, BG at 100 mg/kg [28], and BG-NPs at 45 mg/kg. The control group received an intraperitoneal injection of physiological buffer saline (PBS). On day 17, the animals were sacrificed, and the samples were collected for further analysis. (Fig. 1).

### Sample collection

The mice were anesthetized using isoflurane, and blood was obtained from the retro-orbital plexus. Then, they underwent centrifugation at 3000 rpm for 15 min to separate the serum. This serum was used to assess IgE levels. The bronchoalveolar lavage fluid (BALF) was collected to identify and count infiltrating inflammatory cells. Additionally, lung tissue was collected, and a part of the lung was homogenized in PBS and then subjected to centrifugation at 2500 rpm for 10 min at 4 °C. The resulting supernatant was promptly used to determine the biochemical parameters. The remaining lung tissue was employed for histopathological and molecular investigations.

### Measurement of serum IgE level

Enzyme-linked immunosorbent assay (ELISA) was utilized to assess serum concentrations of IgE using an ELISA plate reader (DAS Instruments, model A3, Rome, Italy) according to manufacturer instructions. The values have been reported as pg/mL.

### Collection of bronchoalveolar lavage fluid (BALF) and cell count

The BALF was obtained using a cannulated trachea and two lavages with one milliliter of PBS. After being collected, the samples were centrifuged for 10 min at 4 °C and 2000 rpm. The cell pellets were reconstituted in 100 μL PBS to conduct total and differential cell counts.

### Oxidative stress biomarkers in the pulmonary tissue

Lung tissue samples were weighed and homogenized, and they were then centrifuged at 25,000 rpm for 10 min at 4 °C, resulting in separation of the supernatant. Subsequently, the levels of glutathione (GSH), superoxide dismutase (SOD), glutathione peroxidase (GPx), malondialdehyde (MDA), and catalase (CAT) were quantified. The MDA was measured following the method of Ohkawa et al. [29], involving the reaction of MDA with Thio barbituric acid (TBA) at a wavelength of 532 nm. Similarly, the evaluation of GSH levels in lung tissue homogenate was conducted based on the procedure of Ellman and Lysko [30] by measuring the resultant compound of the reaction of GSH with 5,5-dithiobis-2-nitrobenzoic acid (DTNB) at 412 nm wavelength of.

The SOD activity was detected using the method outlined by Kakkar et al. [31], which involves assessing the intensity of chromogen in N-butanol at a 560 nm wavelength. Meanwhile, GPx activities were measured following the approach by Rotruck et al. [32], who analyzed the activity of GPx to decompose H2O2 at a wavelength of 412 nm.

Finally, according to Aebi [33], the CAT activity was evaluated by measuring the decomposition of H_2_O_2_ against a wavelength of 240 nm.

### *Iron* levels in lung tissue

Fresh lung tissue was homogenized in 1 mL of PBS. The supernatants were collected after the samples were centrifuged at 10000 g to remove insoluble material. The Iron Assay Kit-Colorimetric (Giza, Egypt) was used to determine the total iron content of mouse lung tissue [34].

### Single-cell gel electrophoresis (Comet assay)

For alkaline comet, lung tissue was homogenized in 1 ml of PBS (Ph 7.4), and clean glass slides were prepared by dipping in 1% normal melting point agarose. Slides were airdried, and then 10 µl of tissue homogenate was mixed with 75 µl of 1% low melting point agarose. The mixture was then smeared on the slides.

After drying, slides were put in lysis buffer (2.5 M NaCl, 10 mM Tris, 100 mM EDTA, 1% Triton X-100, and 10% DMSO, pH 10) for 1 h at 4 °C. They were put in horizontal gel electrophoresis in electrophoresis buffer (300 mM NaOH, 1 mM EDTA, pH13.0) for 30 min. After this, the current was applied (300 mA, 25 V) for 30 min. Following the run, slides were put in a neutralization buffer (0.4 M Tris, pH 7.5) 3 times for 5 min. Finally, the slides were dehydrated in 99.9% ethanol for 2 min. For microscopic examination, slides were stained with 1X ethidium bromide. Microscopic photos were taken using an inverted fluorescence microscope. (Olympus CKX53 microscope, Olympus CX50 camera, cell sense software, Japan). Analysis was carried out using Comet Score 2.0, Tritek. Neutral Comet was applied with the same procedures. The electrophoresis buffer was prepared differently (100 mM Tris and 300 mM sodium acetate at pH 8.5). Fifty scores were recorded for every slide to evaluate DNA damage according to tail length (TL), tail moment (TM), and olive moment (OM) [35].

### Histopathological investigation

#### Hematoxylin and eosin (H&E) staining

The lung tissue was collected from animals and fixated in 10% neutral buffered formalin. Samples were removed from the fixative, washed with PBS, dehydrated in ascending dilutions of ethanol, and then embedded in paraffin wax. Thin sections of about 4 µm were cut from blocks and stained with hematoxylin and eosin (H&E) to evaluate the airways’ morphology and immune cell infiltration [27].

#### Prussian blue stain

Lung tissue sections were dewaxed and rehydrated with distilled water. Slides were then immersed in a solution prepared by combining equal portions of 10% potassium ferrocyanide and 20% hydrochloric acid for 20 min. The slides were then washed three times with distilled water. The slides were counterstained with a nuclear fast red solution for 5 min and cleaned twice in distilled water. After dehydration, the slides were mounted with a resinous mounting media DPX [36].

### Western blot analysis

The protein extraction and purification procedure employed TriFast for simultaneous RNA, DNA, and protein isolation. It initiated with tissue homogenization in TriFast, followed by phase separation using chloroform, which separated RNA into the aqueous phase and DNA and proteins into the interphase. RNA was precipitated with isopropanol, DNA with ethanol, and proteins with isopropanol. Protein pellets underwent washing with guanidinium hydrochloride in ethanol, were solubilized in SDS, and underwent electrophoresis on SDS-PAGE gels. Following gel staining with Coomassie blue, data analysis was performed using a Gel documentation system. For Western blotting, proteins were transferred to a nylon membrane, probed with an anti-GPx4 antibody and β-actin (Abcam, ab231174), and detected with HRP-conjugated secondary antibodies. Blotting buffers, blocking, and antibody solutions were utilized in this process [37].

### Statistical analysis

Statistical analysis was conducted using IBM’s Statistical Package for the Social Sciences (SPSS) version 25. The data were expressed as Mean ± Standard Error of the Mean (SEM). Group comparisons were examined using a one-way analysis of variance (ANOVA). Graphs were generated using GraphPad Prism version 8. Duncan’s post hoc test was employed to compare group means with statistical significance at a threshold of (*P* < 0.05).

## Results

### Characterization of BG and BG-NPs

#### FT-IR analysis

**Fig. 2:**
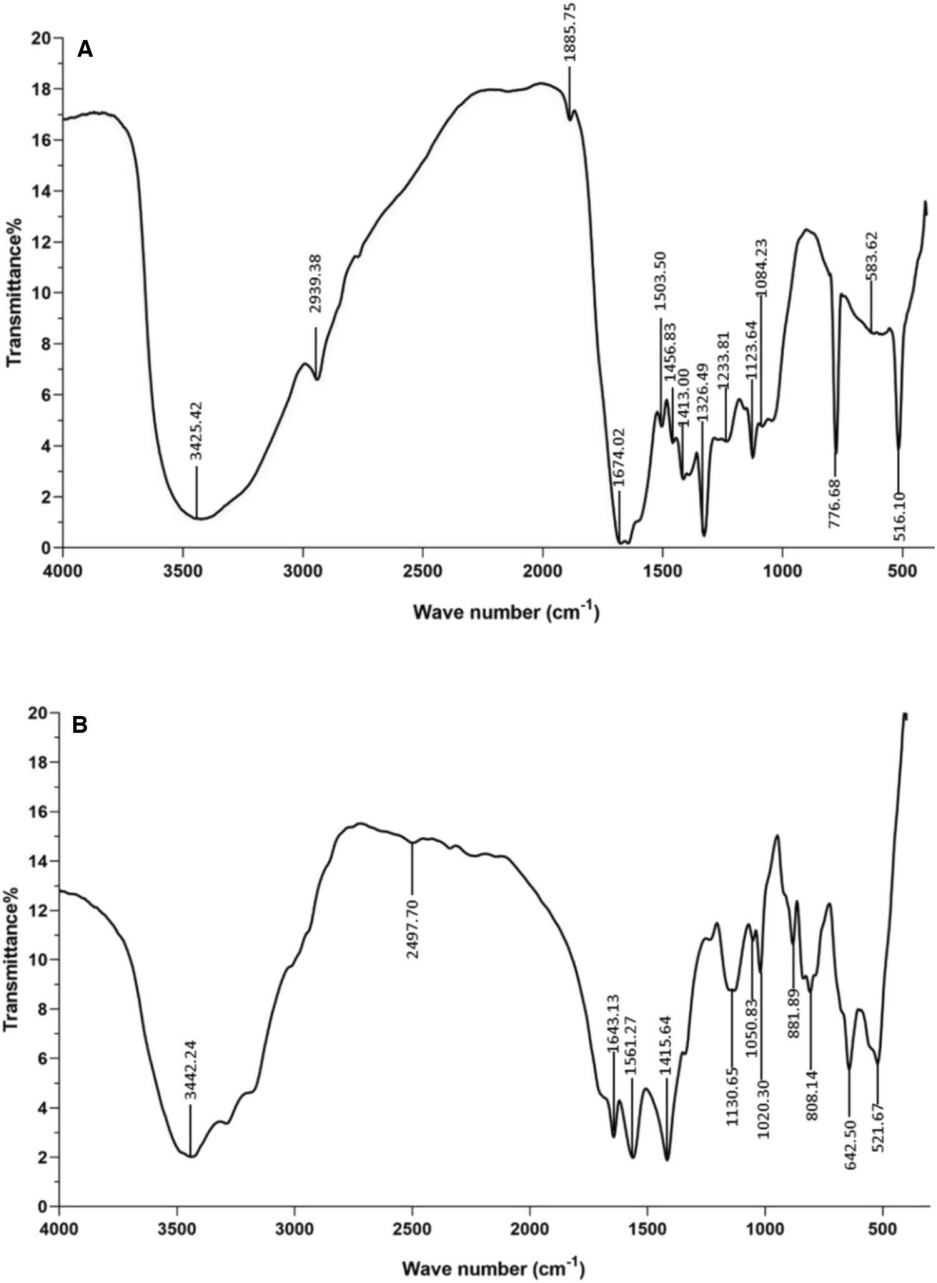
FT-IR spectra of BG and BG-NPs. **A** FT-IR spectrum of BG demonstrating characteristic peaks at 3425.42 cm^−1^, 2939.38 cm-1, 1674.02, 1503.50, and 1084.23 cm^−1^, which attributed to the stretching vibrations of O–H, C–H, C = O, C–H, and C–O functional groups, respectively. **B** The FT-IR spectrum of BG-NPs peaks at 2497.70 cm^−1^, corresponding to phosphate groups

The FT-IR spectrum of BG exhibited several characteristic peaks. Notable peaks were observed at 3425.42, 2939.38, 1885.75, 1674.02, 1503.50, 1456.83, 1413.00, 1326.94, 1233.81, 1123.46, 1084.23, 776.68, 583.62, and 516.10 cm^−1^ (Fig. 2A). The strong peak at 3425.42 cm^−1^ correlates with the stretching vibrations of O–H bonds, while the peaks at 2939.38 cm-1 are attributed to C–H bonds. Peaks at 1674.02, 1503.50, and 1084.23 cm^−1^ are associated with C = O, C–H, and C–O functional groups, respectively. These FT-IR spectral characteristics are consistent with the presence of BG in the sample. The FT-IR spectrum of BG-NPs peaked at 2497.70 cm^−1^, which can be ascribed to the OH-P = O stretch (Fig. 2B). This finding suggests that BG nanoparticle synthesis led to incorporating phosphate groups.

#### UV/Vis analysis

**Fig. 3:**
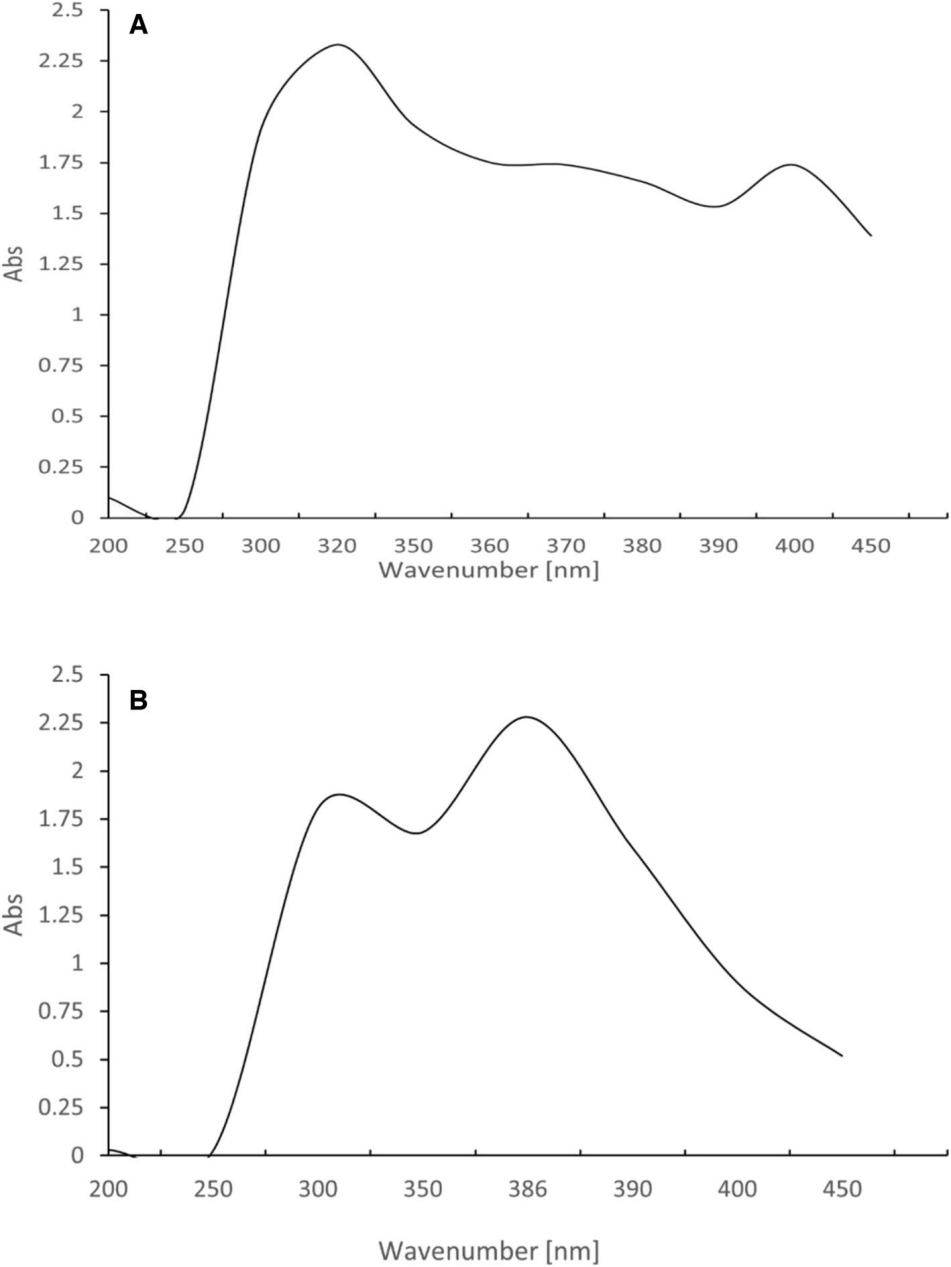
UV/Vis. Spectrum of BG and BG-NPs. **A** BG showed a strong peak at 310 nm. **B** BG-NPs showed a strong peak at 386 nm

The UV/Vis. Analysis of BG revealed a prominent peak at 310 nm. At the same time, BG-NPs revealed a prominent peak at 386 nm, indicating the stability of BG nanoparticles at room temperature and the presence of BG-NPs (Fig. 3) [25].

#### XRD analysis

**Fig. 4:**
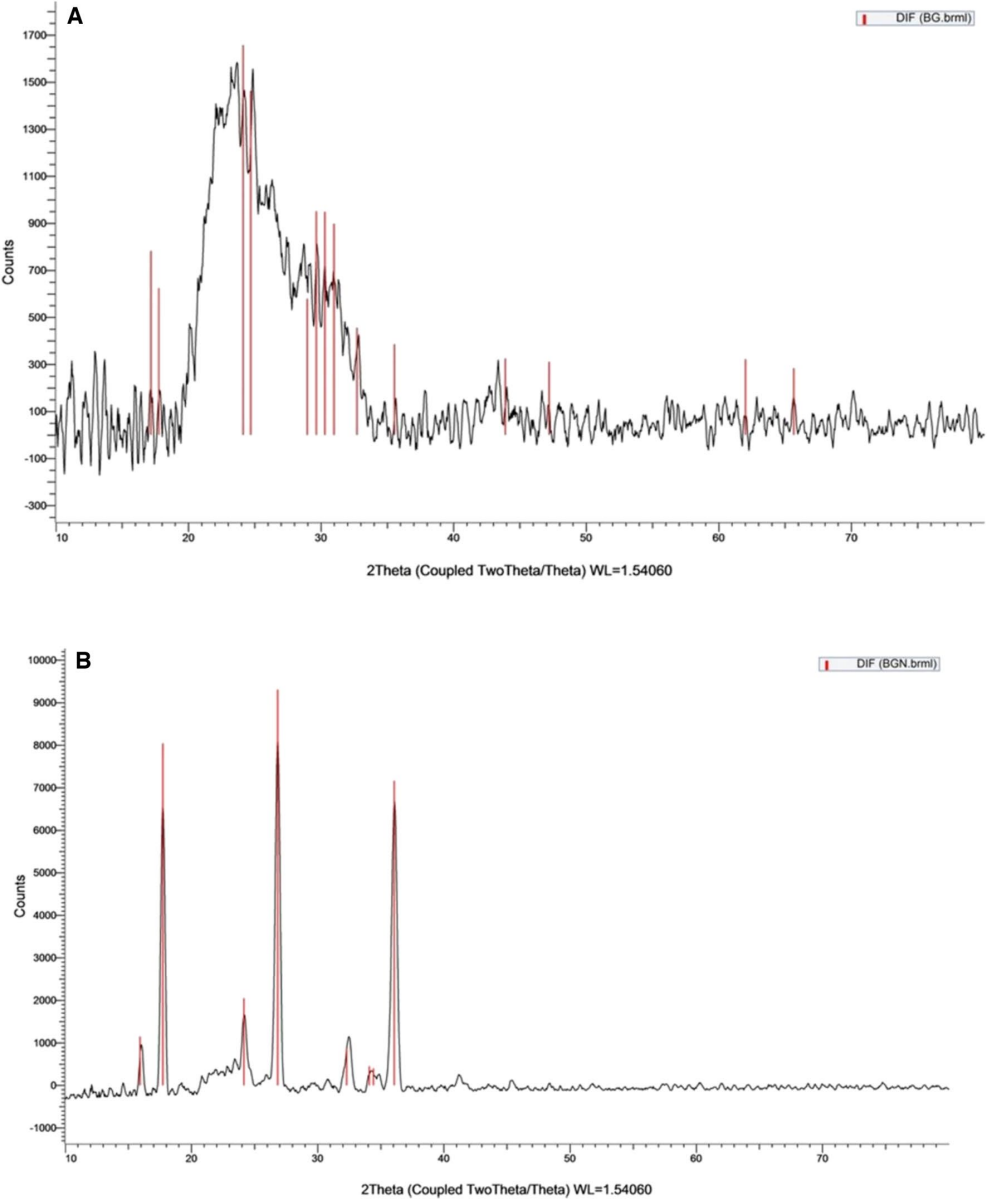
X-ray diffraction (XRD) curves of **A** BG and **B** BG-NPs

X-ray powder diffraction patterns of BG and BG-NPs are shown in (Fig. 4). The diffraction peak of BG is at around 17.185° and 65.658° of 2*θ*. XRD patterns of BG-NPs show a diffractive peak in the region from 2*θ* = 15.930 to 36.070°. The BG-NPs peaks corresponded to 2*θ* = 17.741°, 26.841°, 36.070°, which were planes of crystalline nature of the BG-NPs and were under the earlier reports on polysaccharides [38, 39].

##### TEM

**Fig. 5:**
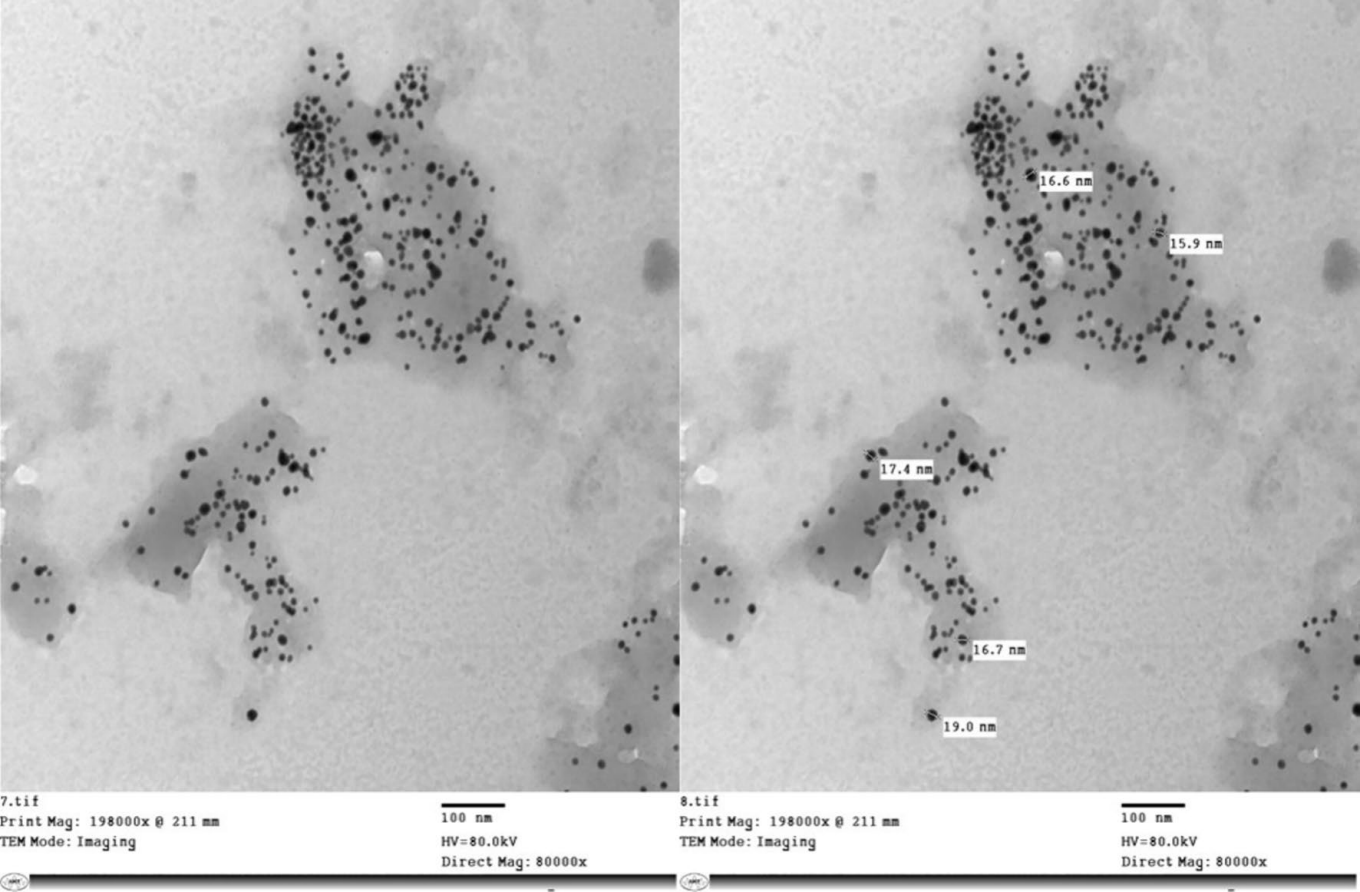
TEM image of BG-NPs showing a uniform size of about 20 nm and spherical shape

TEM imaging revealed that BG-NPs exhibited a spherical shape and uniform size, with an average diameter of approximately 20 nm (Fig. 5). The uniformity in size indicates the successful synthesis of BG nanoparticles with controlled morphology.

#### Zeta potential and DLS analysis

**Fig. 6:**
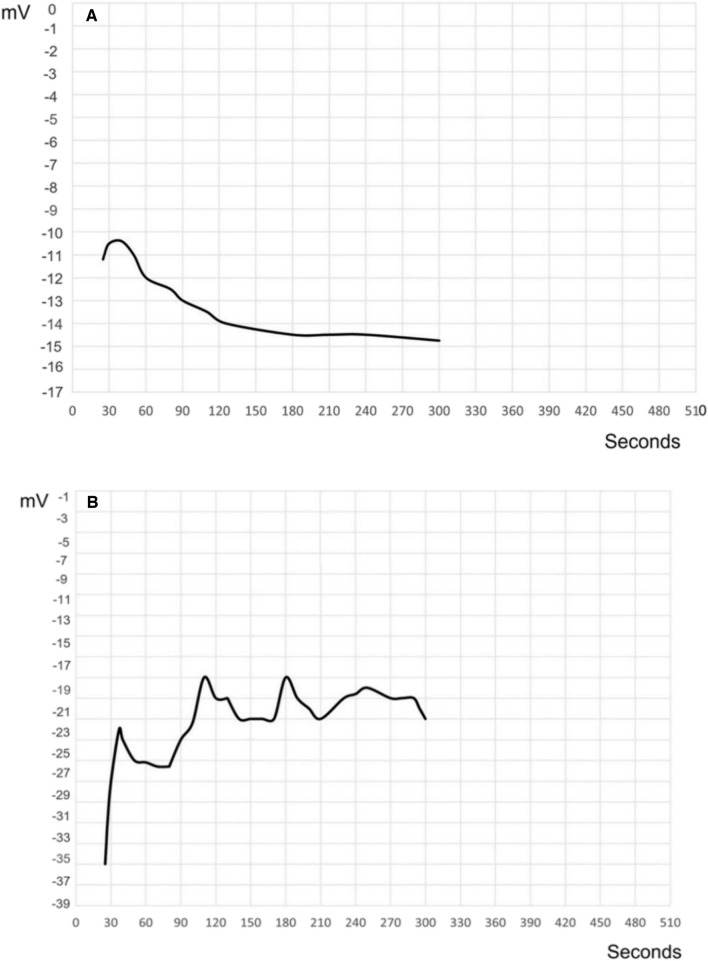
Zeta potential analysis of BG and BG-NPs. **A** Zeta potential analysis of BG demonstrated a negative value of -14.51 mV. **B** Zeta potential analysis of BG-NPs demonstrated a negative shift to -20.67 mV

The zeta potential analysis of BG indicated a negative value of -14.51 mV (Fig. 6A), highlighting the stability of BG particles. BG-NPs exhibited a further negative shift in zeta potential to -20.67 mV (Fig. 6B). This shift may be attributed to the interaction of BG-NPs with TPP [25], suggesting the successful functionalization of nanoparticles.

**Fig. 7:**
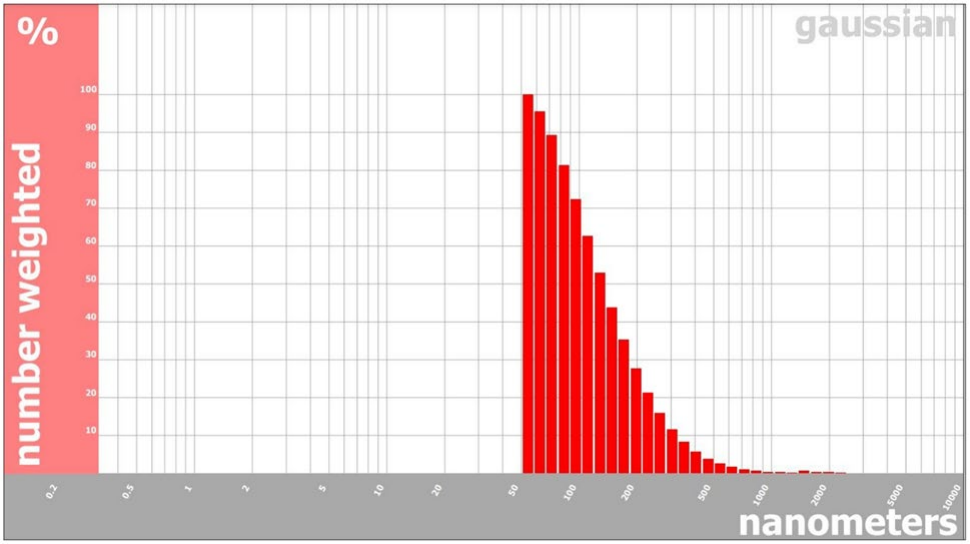
Particle size analysis of BG-NPs showing a size of 68.28 nm and PI of 0.84

Dynamic light scattering (DLS) analysis revealed that BG-NPs had a particle size of 68.28 nm with a polydispersity index (PI) of 0.84 (Fig. 7). The relatively narrow size distribution and the PI value close to 1 indicate the stability and uniformity of the synthesized nanoparticles.

### Effect of BG-NPs on serum IgE levels

**Fig. 8:**
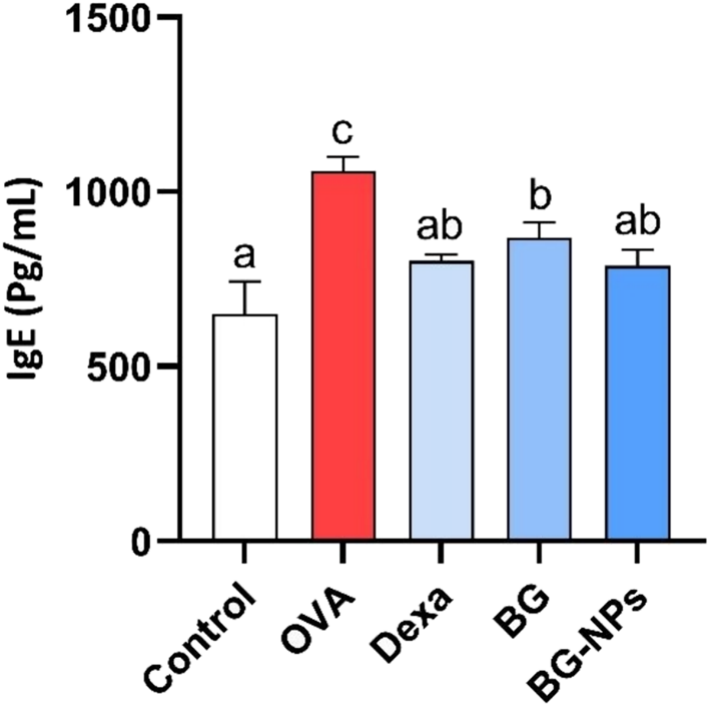
Effect of BG-NPs on the serum levels of IgE. Values are given as a mean for each group ± standard error of the mean (SEM). The value with different superscript letters is significantly different (*P* < 0.05)

The key characteristic of allergic asthma is a rise in serum IgE levels. In our investigation, OVA-induced asthmatic mice’s blood IgE levels were significantly higher (*P* < 0.05) than those of control mice. IgE production was significantly decreased (*P* < 0.05) by intraperitoneal injections of BG (100 mg/kg), Dexa (1 mg/kg), and BG-NPs (45 mg/kg) (Fig. 8).

### Effect of BG-NPs on airway-infiltrating inflammatory leukocytes

**Fig. 9:**
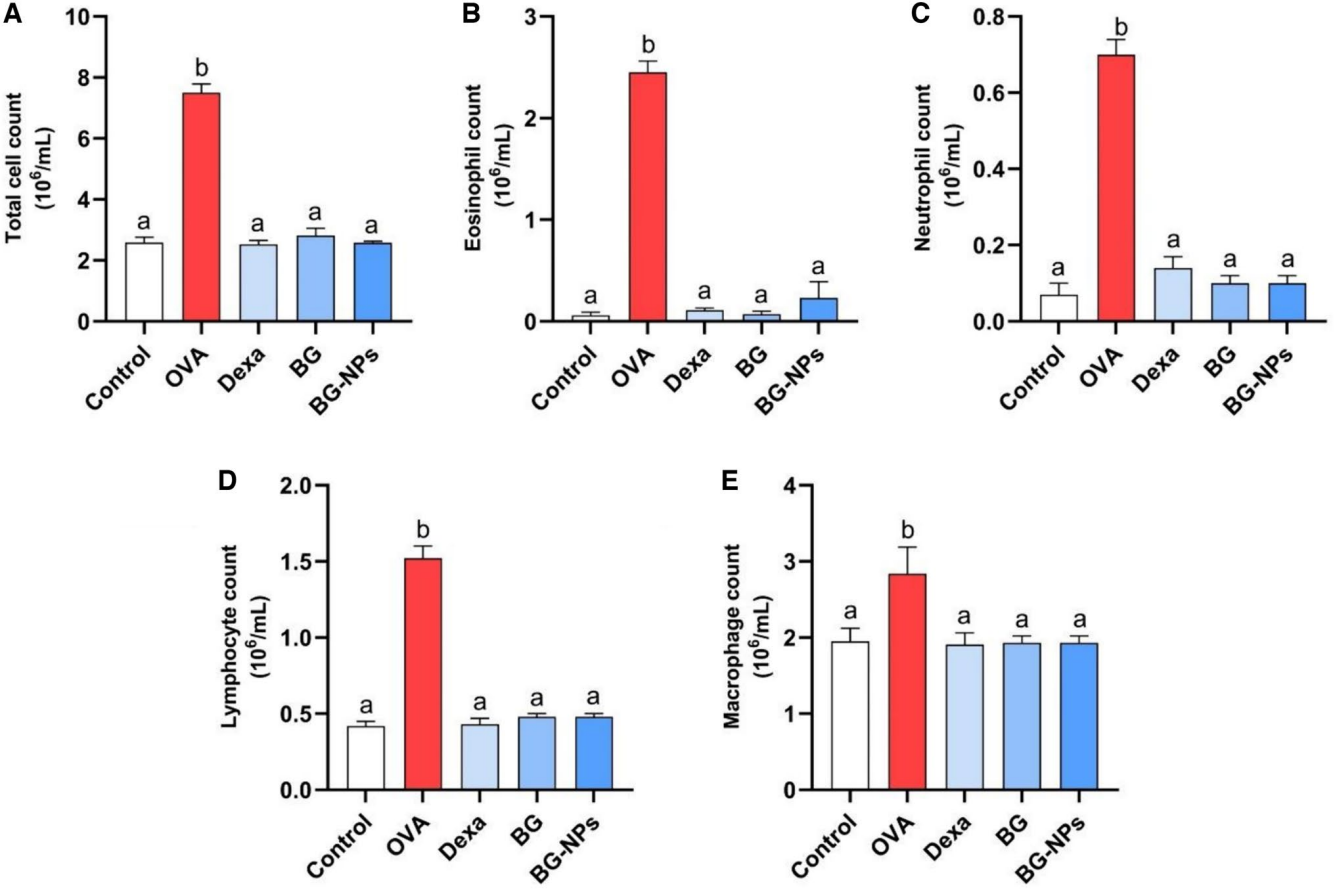
Effect of BG-NPs on inflammatory cell count in BALF of asthmatic mice. **A** Total cell count, **B** Eosinophil count, **C** Neutrophil count, **D** Lymphocyte count, and **E** Macrophage count. Values are given as means for 8 mice in each group ± standard error of the mean (SEM). The value with different superscript letters is significantly different (*P* < 0.05)

In the BALF of OVA-induced asthma mouse models, the inflammatory cell counts were examined to assess the suppressive effect of BG-NPs on the infiltration of inflammatory cells (Fig. 9). As predicted, the OVA group exhibited significantly (*P* < 0.05) higher levels of inflammatory cells in BALF compared to the control group. This infiltration was reduced (*P* < 0.05) by treatment with BG (100 mg/kg), BG-NPs (45 mg/kg), and DEX (1 mg/kg).

#### Effect of BG-NPs on oxidative stress biomarkers in the lung tissue

**Fig. 10:**
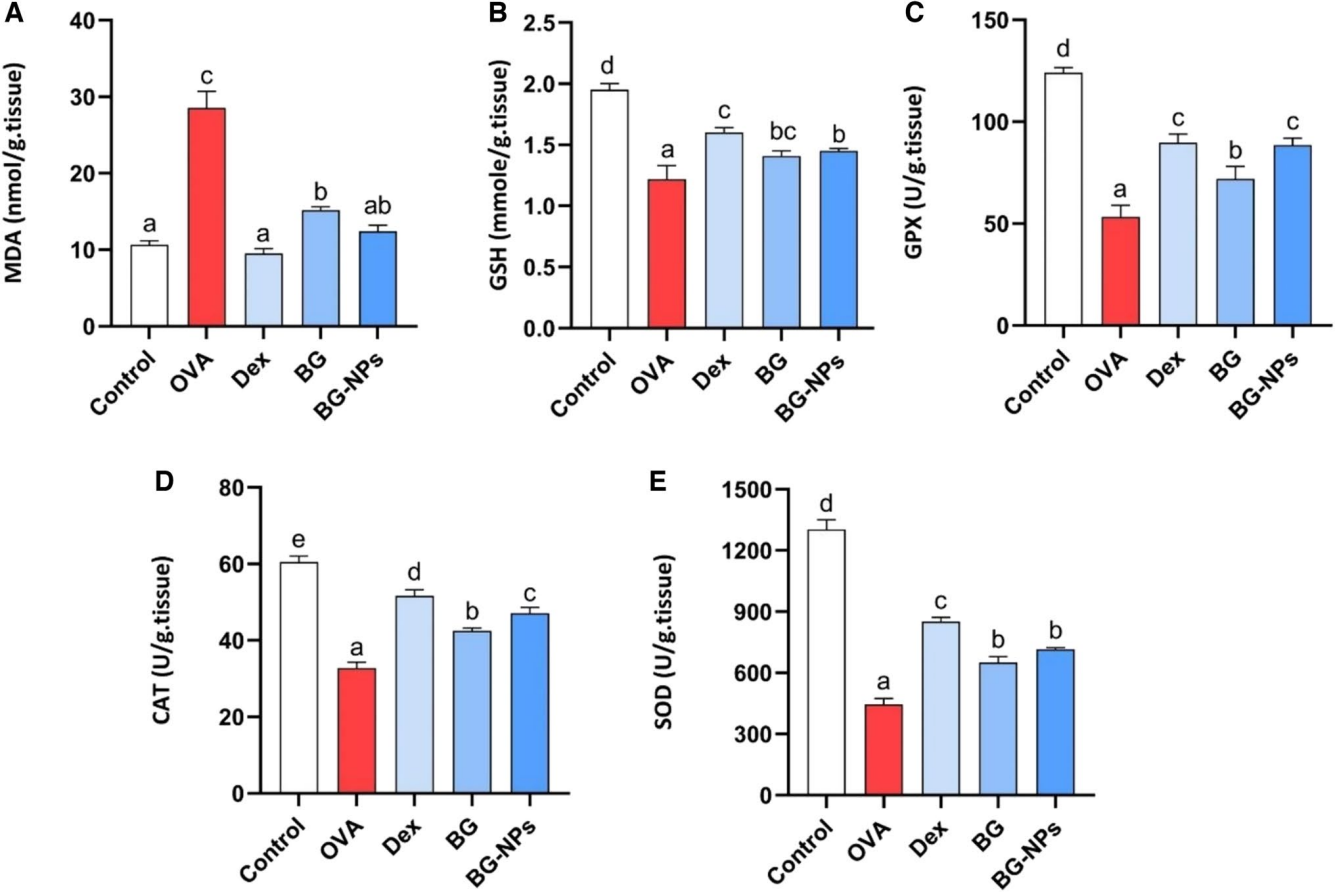
displays data regarding lung oxidative stress biomarkers (MDA, GSH, CAT, GPx, and SOD). The MDA concentrations in OVA mice were significantly higher than those in the control group (P < 0.05). On the other hand, groups treated with Dexa, BG, and BG-NPs exhibited significant reductions (P < 0.05) with percentages 66.71%, 56.51%, and 46.779%, Furthermore, asthmatic mice showed a significant (P < 0.05) decrease in GSH levels as well as in the activities of GPx, SOD, and CAT. Despite this, mice treated with Dexa, BG, and BG-NPs significantly increased (P < 0.05) with percentages of 31.46%, 18.87%, and 15.59% for GSH, 68.29%, 66.10%, and 34.90% for GPx, 90.89%, 60.36%, and 45.90% for SOD, and 57.60%, 43.77%, and 29.84% for CAT

Figure 10 displays data regarding lung oxidative stress biomarkers (MDA, GSH, CAT, GPx, and SOD). The MDA concentrations in OVA mice were significantly higher than those in the control group (P < 0.05). On the other hand, groups treated with Dexa, BG, and BG-NPs exhibited significant reductions (P < 0.05). Furthermore, asthmatic mice showed a significant (P < 0.05) decrease in GSH levels as well as in the activities of GPx, SOD, and CAT. Despite this, mice treated with Dexa, BG, and BG-NPs significantly increased (P < 0.05).

### Effect of BG-NPs on total *iron* content in lung tissue

**Fig. 11:**
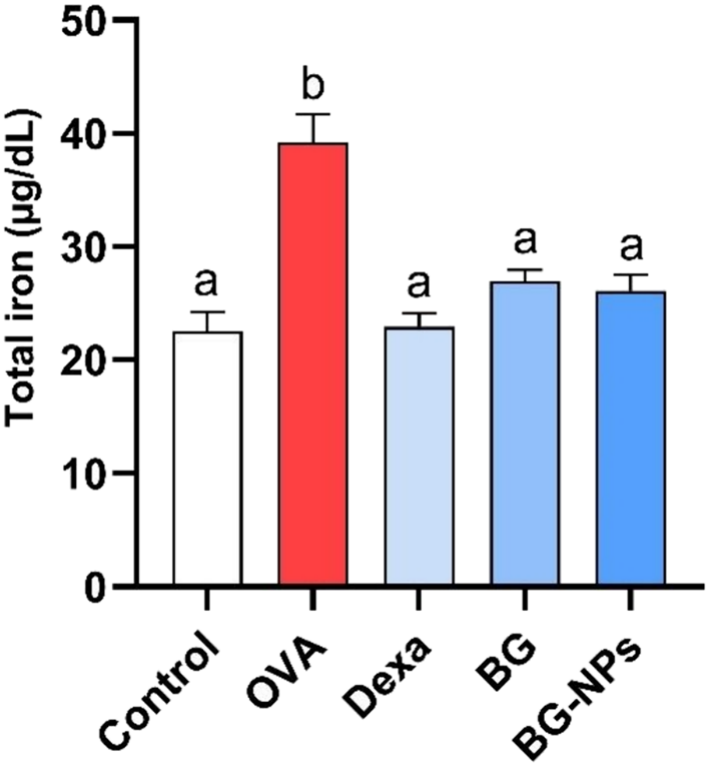
Effect of BG-NPs on the total iron levels in lung tissue. Values are given as a mean for 8 mice in each group ± standard error of the mean (SEM). The values with different superscript letters differ significantly (*P* < 0.05)

We investigated how BG-NPs affected the amount of iron accumulated in asthmatic mice’s lung tissue. In our study, the total iron levels of OVA-induced asthmatic mice were significantly greater (*P* < 0.05) than those of control mice. Intraperitoneal injections of BG (100 mg/kg), Dexa (1 mg/kg), and BG-NPs (45 mg/kg) significantly (P < 0.05) reduced the iron concentration (Fig. 11). These findings implied that treating asthmatic mice with BG-NPs decreased the amount of iron deposited in their lung tissues.

### BG-NPs inhibited DNA damage in the lung cells

**Fig. 12:**
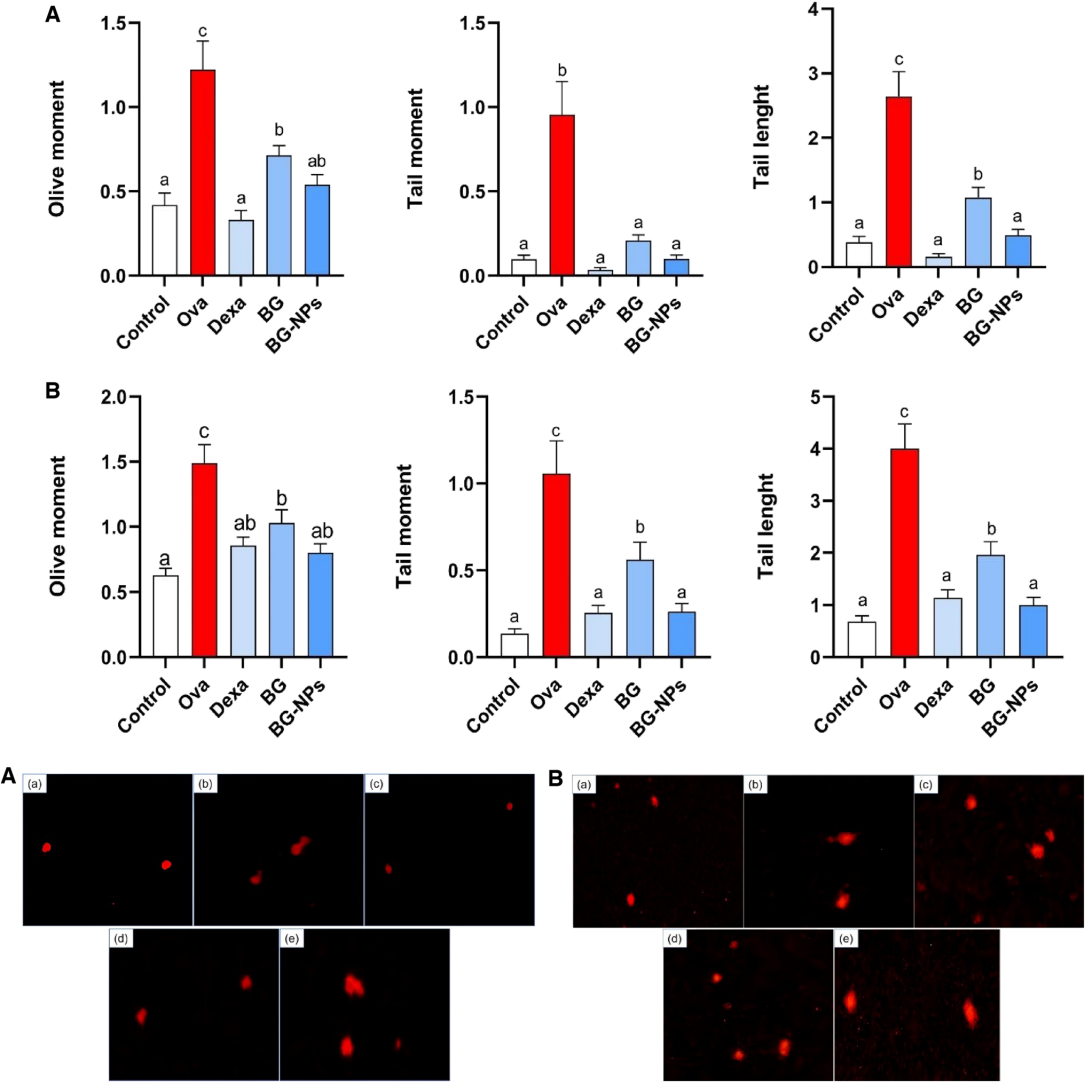
Quantifying the treatment groups’ DNA damage parameters (olive moment, tail moment, and tail length). At least 50 cells were evaluated per sample. CometScore (V2.0) software was utilized to evaluate DNA damage indicators **A** Alkaline Comet assay. **B** Neutral Comet assay. Values are given as means for 8 mice in each group ± standard error of the mean (SEM). The values with different superscript letters differ significantly (*P* < 0.05)

The alkaline comet assay assessed DNA damage induced by oxidative stress. The OVA group exhibited significant DNA damage, while both BG and BG-NPs showed minimal DNA damage compared to the OVA group (Fig. 12A). The parameters measured, including tail length (TL), tail moment (TM), and olive moment (OM), all demonstrated a significant difference between treated groups with Dexa, BG, and BG-NP and the OVA group (*p* < 0.05) (Table 1). The neutral comet assay evaluated double-strand DNA damage (apoptotic DNA damage). TL, TM, and OM were measured to assess and compare apoptotic DNA damage between groups (Table 2). The results revealed that BG and BG-NPs significantly (*p* < 0.05) reduced apoptotic DNA damage compared to the OVA group regarding TL, TM, and OM parameters (Fig. 12B). Additionally, Tables 1 and 2 display the percentage of change of the treated groups compared to the OVA group in the alkaline and neutral comet assay, respectively.

**Table 1:** Percentage Comparison of Alkaline Comet Assay Parameters Between Treated Groups and the OVA Control Group

	Tail length	Tail moment	Olive moment
Dexa	− 94.4%	− 96.77%	− 74.70%
BG	− 82.51%	− 90.56%	− 58.68%
BG-NPs	− 61.53%	− 80.15%	− 45.34%

**Table 2:** Percentage Comparison of Alkaline Comet Assay Parameters Between Treated Groups and the OVA Control Group

	Tail length	Tail moment	Olive moment
Dexa	− 71.5%	− 75.78%	− 42.51%
BG	− 51%	− 46.85%	− 30.85%
BG-NPs	− 75%	− 75.21%	− 46.24%

#### BG-NPs ameliorated histopathological changes in lung tissue

#### H&E

**Fig. 13:**
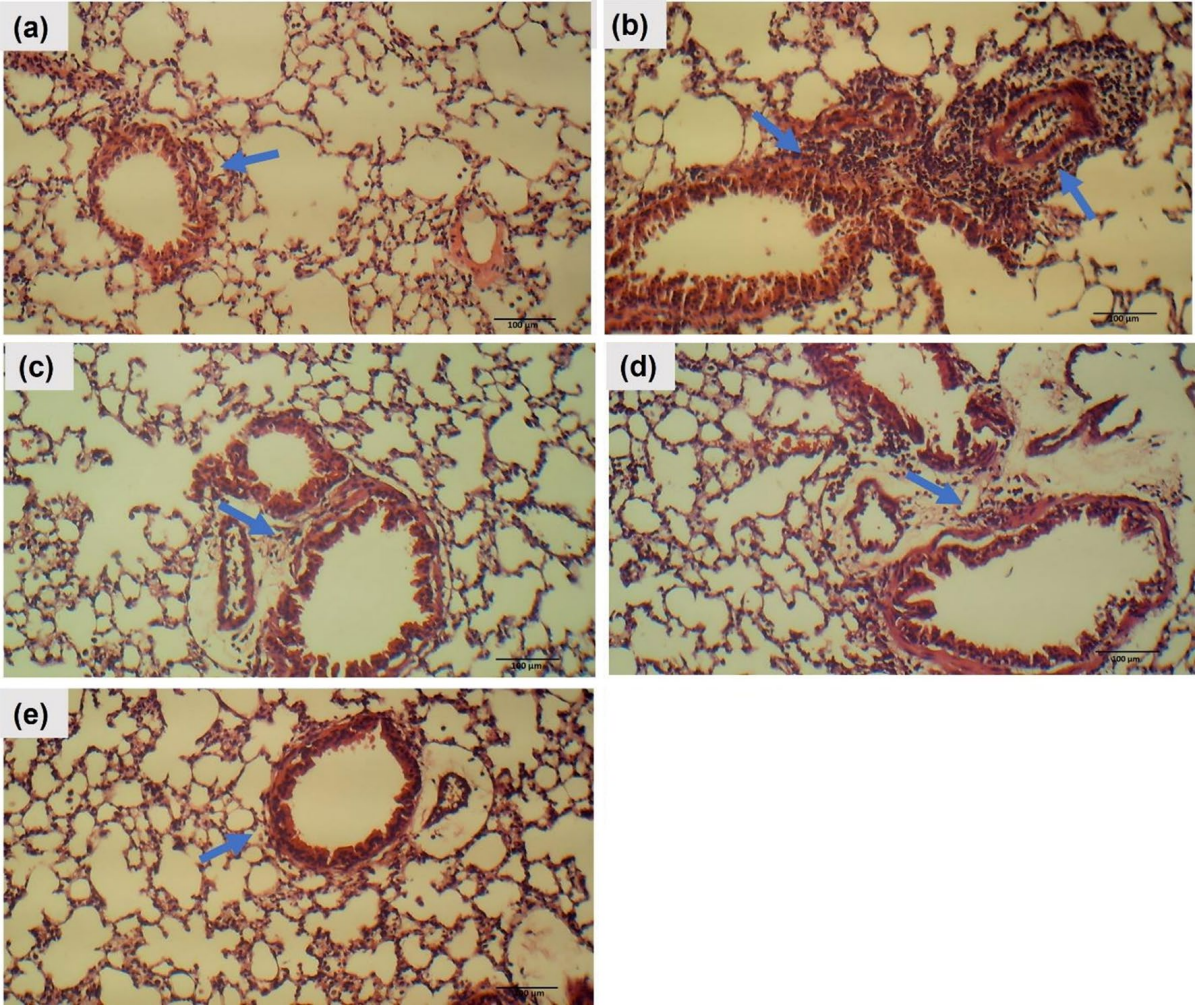
Effect of BG-NPs on histological alterations of the lung tissue from mice of different groups. (H&E, Magnification: X100). **a** Control, **b** OVA, **c** Dexa-treated, **d** BG-treated, and **e** BG-NPs-treated groups. (Blue arrows indicate peri-bronchial and perivascular inflammatory cells)

The control mice showed a normal histological appearance of the lung tissue with minimal infiltration of inflammatory cells. On the other hand, the OVA group showed excessive infiltration of inflammatory leukocytes in the lung tissues’ peri-bronchial and peri-vascular regions. The treatment groups significantly attenuated the infiltration of the inflammatory cells. (Fig. 13).

#### Prussian blue

**Fig. 14:**
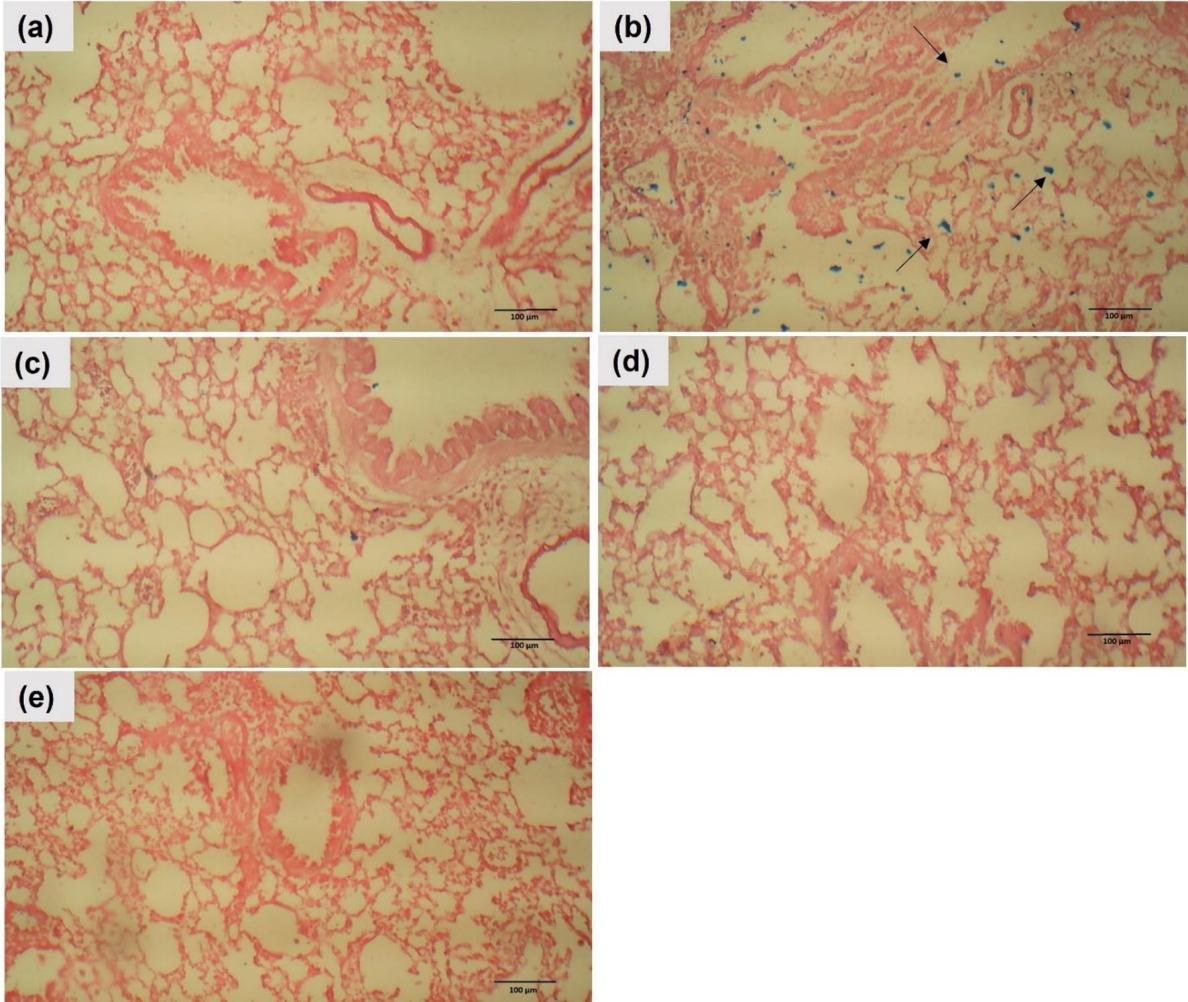
Effect of BG-NPs on iron accumulation in the lung tissue from mice of different groups. (Prussian blue, Magnification: X100). **a** Control, **b** OVA, **c** Dexa-treated, **d** BG-treated, and **e** BG-NPs-treated groups

Prussian blue was used to detect iron accumulation in the lung tissue. The control group showed no iron accumulation. On the other hand, the OVA group exhibited increased levels of iron. However, the intraperitoneal injection of Dexa (100 mg/kg), BG (100 mg/kg), and BG-NPs (45 mg/kg) significantly reduced the levels of iron in the lung tissue (Fig. 14).

### Effect of BG-NPs on GPx4 expression in lung tissue

**Fig. 15:**
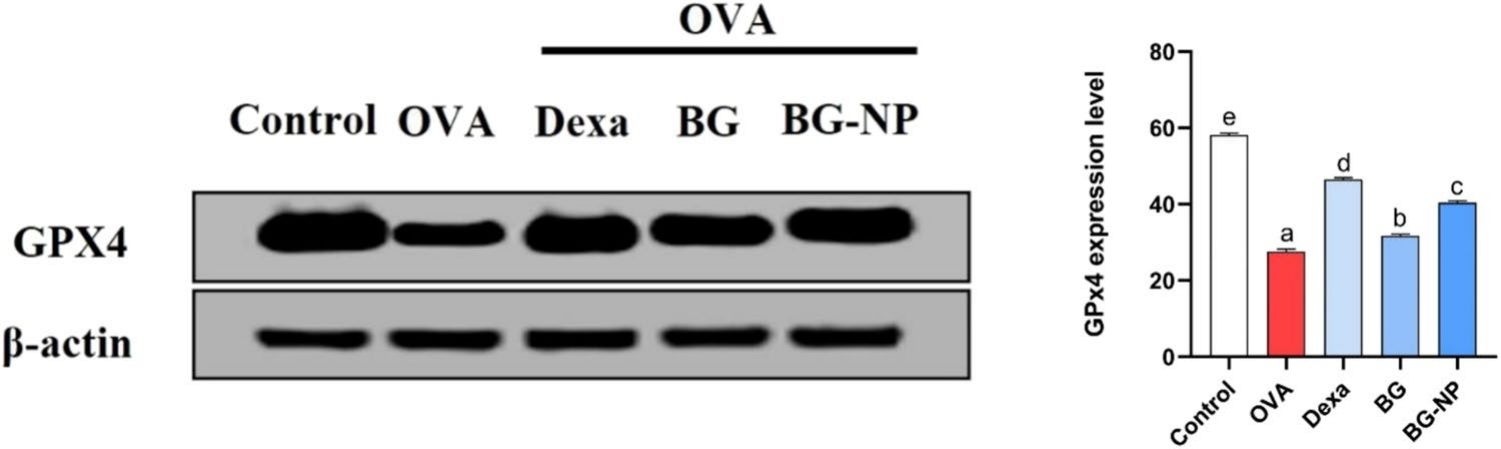
The effect of BG-NPs on lung tissue expression of GPx4. Western blot was used to measure the amount of GPx4 protein in the lung with anti-β-actin as a loading control. The values are expressed as the means ± standard error of the mean (SEM) for 8 mice in each group. The values with different superscript letters differ significantly (*P* < 0.05)

Since it has been suggested that GPx4 is a crucial regulator of ferroptosis, we looked for protein expression levels. Our results showed that, in comparison to the control group, OVA exposure downregulated the protein levels of GPx4. However, intraperitoneal injection of Dexa (100 mg/kg), BG (100 mg/kg), and BG-NPs (45 mg/kg) significantly increased (*P* < 0.05) the expression of GPx4 (Fig. 15).

## Discussion

Asthma is a persistent inflammatory respiratory condition that affects the airways. The current investigation utilized a mouse model of allergic asthma to assess the impact of BG-NPs on IgE production, lung-infiltrating inflammatory cells, oxidative stress markers, DNA damage, airway remodeling, and GPx4 expression.

Elevated blood IgE levels are the hallmark of allergic asthma [40]. The current study demonstrates a notable rise in serum IgE levels, which indicates the allergic inflammation brought on by exposure to OVA [41]. Similarly, serum and BALF IgE content have consistently increased in experimental animal models of allergic asthma produced by OVA [42, 43]. Conversely, the group that received BG-NP treatment had noticeably decreased serum IgE levels. These findings align with earlier research where BG exhibited an antiallergic effect by inhibiting IgE production [44]. This outcome demonstrates the anti-allergic capacity of BG-NPs by preventing the increase in serum IgE concentration induced by OVA.

A major influx of inflammatory cells is commonly regarded as the primary event during the pathogenesis of allergic asthma [45]. The excessive occurrence of inflammatory cells produces pro-inflammatory chemicals, eventually leading to severe pulmonary injury [46]. The current study showed that the OVA-induced mice had markedly higher total and differential BALF immune cells. These findings are consistent with prior research demonstrating that OVA exposure significantly increases the influx of inflammatory cells into the airways [47]. The elevated cell counts indicate allergic inflammation [48]. In contrast, BG-NPs significantly decreased the number of inflammatory cells infiltrating the airway in the BALF. The findings of this study strongly provide persuasive support for the concept that BG is effective in preventing allergic inflammatory reactions [49]. This highlights the anti-inflammatory potential of BG-NPs in the context of allergic asthma.

The histopathological analysis of the lung tissues has confirmed the airway-infiltrating inflammatory cell count results. In OVA-challenged mice, significant influxes of inflammatory cells entered the airway and encircled the blood vessels. BG-NPs effectively decreased lung inflammation and inflammatory cell infiltration; these effects were consistent with the cell counts in BALF. These findings are consistent with previous research where BG treatment attenuated the infiltration of inflammatory cells into the airways [28]. These results verify that BG-NPs’ protective effect against OVA-induced allergic asthma is associated with reducing the number of inflammatory cells in the lung tissues.

Further, for exploration of DNA damage in asthma, we use Comet assay, alkaline comet to evaluate alkaline DNA damage in single strand (single-strand breaks, basic sites, and alkali-sensitive sites), the neutral to evaluate the double strand Damage (apoptosis DNA damage) [50, 51]. In our study, the OVA-challenged mice showed DNA damage in the lung tissue. The oxidative DNA damage in the previous investigation showed a significant increase in the asthma model [52]; in our study, BG and BG-NPs decreased DNA damage (alkaline and neutral) by showing a significant decrease in TL, TM, and OM.

Oxidative stress is a major factor in the pathogenesis of asthma [53]. Excessive ROS is released by activated inflammatory leukocytes and respiratory epithelial cells, causing oxidative injury in the airway [54]. MDA is regarded as a peroxidation byproduct that results from ROS affecting lipids and indirectly indicates oxidative stress [55]. According to this study, the MDA levels in the OVA group significantly increased. Our findings align with previous research where OVA exposure increased the MDA concentration [56]. Meanwhile, the groups given Dexa, BG, and BG-NPs decreased noticeably. These findings support a previous study in which BG treatment significantly reduced the amount of MDA in septic rats [57]. GSH and CAT are necessary antioxidants to reduce airway cell destruction and fibrosis [58]. According to this study, the OVA group’s GSH levels and CAT activity significantly decreased. Our findings support earlier research showing OVA exposure decreased CAT and GSH levels [59, 60]. Nonetheless, there was an evident rise in the groups that received BG, BG-NPs, and Dexa. These findings support earlier research showing that BG treatment significantly raised GSH and CAT levels [61, 62]. SOD and GPx are critical for decreasing oxidative stress. SOD dismutates superoxide (O^2−^) to produce oxygen (O_2_) and peroxide (H_2_O_2_). GPx also preserves the cell’s membrane organization, breaks down H_2_O_2_, and protects physiological systems from oxidative harm [63]. In this study, OVA-challenged mice had significantly lower SOD and GPx activity. Our findings support previous findings that OVA inhalation reduced SOD and GPx activity [64]. Groups obtaining BG, BG-NPs, and Dexa increased significantly. These findings support a previous investigation demonstrating that BG treatment greatly increased SOD and GPx contents [65].

Ferroptosis, a type of programmed cell death, results from too much build-up of lipid peroxides and iron-dependent ROS [66]. Elevated ferroptosis in asthma is brought on by oxidative stress, and airway inflammation is brought on by elevated iron levels [67]. When intracellular iron builds up, glutathione is depleted, GPx4 is inactivated, lipid peroxidation is elevated, and ferroptosis starts and intensifies [68]. According to this study, ferroptosis has a role in the pathophysiology of OVA-induced asthma, as evidenced by the elevation of iron, oxidative DNA damage, and MDA levels and the downregulation of the antioxidant protein GPx4. However, following the administration of Dexa, BG, and BG-NPs, there was a significant reduction in the levels of MDA and iron and a notable increase in the expression of GPx4 protein.

**Fig. 16:**
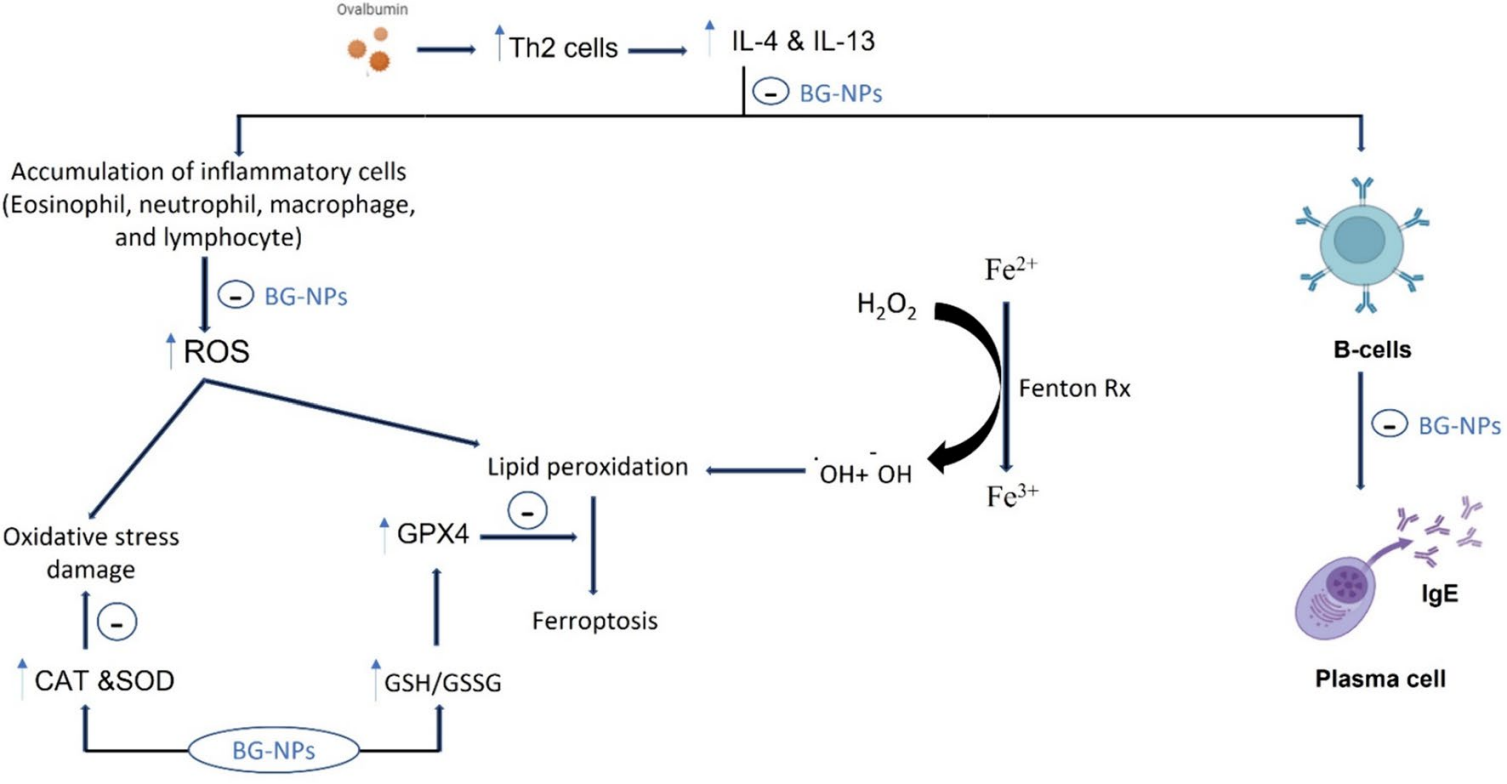
Mechanism of action of BG-NPs

The results, as shown in Figure, show that BG-NPs treatment effectively protects the lungs from oxidative stress and inflammation. This protection is essentially based on maintaining an appropriate balance between ROS and antioxidants, lowering IgE and Th2-related cytokine production, and inhibiting inflammatory immune cell infiltration. Furthermore, BG-NPs treatment lowers the amount of iron in lung tissue. These findings indicate that it may be effective against ferroptosis in allergic asthma (Fig. 16).

## Conclusion

In summary, BG-NPs exhibited anti-asthmatic effects, including reductions in airway inflammation, IgE production, DNA damage, and oxidative stress. Additionally, BG-NPs reduced ferroptosis by inhibiting iron accumulation and upregulating GPx4 protein levels in the lung tissue, offering an innovative strategy for the clinical management of asthma. The results demonstrated that BG-NPs may help treat allergic asthma and other allergy-mediated diseases.

## Data Availability

The data that support the findings of this study are available from the corresponding author upon reasonable request.
